# Influence of Different Load Conditions on Lower Extremity Biomechanics during the Lunge Squat in Novice Men

**DOI:** 10.3390/bioengineering9070272

**Published:** 2022-06-22

**Authors:** Lidong Gao, Zhenghui Lu, Minjun Liang, Julien S. Baker, Yaodong Gu

**Affiliations:** 1 Faculty of Sports Science, Ningbo University, Ningbo 315211, China; gaolidong1997@hotmail.com (L.G.); luzhenghui_nbu@foxmail.com (Z.L.); 2 Department of Physical and Health Education, Udon Thani Rajabhat University, Udon Thani 41000, Thailand; 3 Center for Health and Exercise Science Research, Department of Sport and Physical Education, Hong Kong Baptist University, Hong Kong; jsbaker@hkbu.edu.hk; 4 Savaria Institute of Technology, Eötvös Loránd University, H-9700 Szombathely, Hungary

**Keywords:** lunge squat, kinematic, kinetics, weight bearing, OpenSim

## Abstract

Objective: The lunge squat is one of the exercises to strengthen the lower limbs, however, there is little evidence of the effects of different equipment. The purpose of this study was to investigate the biomechanical effects of different types of equipment and loads on the lunge squat’s effect on the lower limbs. Methods: Fourteen male fitness novices participated in the experiment. Kinematics and kinetics in the sagittal plane using dumbbells, barbells, and weighted vests were measured using OpenSim. Two-way repeated measures ANOVA and one-dimensional statistical parametric mapping were used in the statistical analysis (SPM1D). Results: Range of motion (ROM) change in the knee joint was more obvious when using a barbell, whereas ROM when using a dumbbell was minimal. Compared to other joints, the joint moment at the hip joint was the largest and changed more significantly with increasing weight-bearing intensity, and the change was more pronounced with the dumbbell. For the center of pressure (COP) overall displacement, the dumbbell produced a smaller range of displacement. Conclusions: Dumbbells are suggested for male beginners to improve stability, barbells for the more experienced, and a low-weighted vest may be more appropriate for those with knee pain.

## 1. Introduction

The lunge squat is becoming increasingly popular as a strength training exercise because of its safety and feasibility [1,2]. Performing the lunge squat requires both feet to be placed on the ground. This can also be a suitable exercise for people with poor lower limb balance [3]. The lunge squat is also a common position used in many sports, and a suitable lunge training program is important to improve athletic performance and rehabilitation after surgery [4].

As a closed-chain exercise modality [4,5,6,7], the lunge squat is performed by mobilizing multiple joints in a coordinated movement. It improves body coordination and has a positive effect on the training of the lower limb and hip muscles [8]. The lunge squat also plays a key role in rehabilitation exercises used for static and dynamic joint balance [1,8]. Therefore, the action is often used in clinical settings, such as rehabilitation following anterior cruciate ligament (ACL) reconstruction surgery [4,9] and as an exercise prescription for fall prevention exercises [2]. Furthermore, because of the strengths and weaknesses in these areas that have been associated with injury development [10,11,12,13], postoperative recovery of the hip and knee joints should be given particular priority. The strength and endurance of the hip muscles, on the other hand, are critical for injury prevention, gait correction, pain alleviation, and improved athletic performance [14,15,16,17]. For example, strengthening the gluteus medius has been proven to be helpful in functional recovery and pain reduction in patients after knee meniscus surgery [15]. In addition, maintaining the gluteus maximus muscle helps reduce the incidence of low back pain and disability [16]. Because of the better training effect on the large muscle groups of the lower limbs, the lunge squat is an exercise that requires physical strength and muscular endurance [18]. It is worth noting that the lunge squat effectively increases the strength of the hip, knee, and ankle joints, as well as the quadriceps [8,19]. In addition, increasing the muscle activity ratio between the medial femoral and lateral femoral muscles through effective exercise maintains strength balance between the right and left muscles around the knee joint [8,20]. A study demonstrated that the lunge effectively improves muscle strength and balance in the lower limbs [3]. The hip muscles’ activation degree significantly affects the ability of the quadriceps and leg muscles to generate force or resist impact during jumping [21]. Additionally, improved hip muscle function may help to prevent common lower limb problems such as anterior cruciate ligament injuries [6]. Farrokhi et al. [22] found that when performing a forward lunge with a more forward-leaning trunk, the extensor impulse and electromyography of the hip joint were significantly increased, PFA was greater than in upright lunges, and hip extensors were enhanced more in forward trunk lunges.

Adding greater weights is the most popular and important method of resistance training for athletes to improve athletic performance [23]. Compared to the ankle and hip joints, it was found that the lunge squat relies more on the knee joints. In addition, the increased external weight causes more mechanical work to be performed at the hip and ankle joints [9]. At the same time, differences in body posture and changes in stride length can affect the biomechanical changes of each joint during the lunge squat. Previous studies have found that increasing the dominant leg’s tibia angle to the ground resulted in a smaller range of motion (ROM) in the anterior knee and a larger ROM in the posterior knee and hip. Meanwhile, as the tibial angle increases, the flexion moment of the anterior leg knee and anterior leg hip decreases, but the flexion moment of the anterior hip joint increases as stride length increases [24]. It has also been observed that muscle activation was significantly higher in the weight-bearing condition than in the self-weight condition when only using barbells, dumbbells, weighted vests, and kettlebells. Quadriceps and hamstring activity were significantly higher in the lunge-step exercise than in the deep squat. However, Wu et al. [4] argued that muscle activation was not affected by changes in weight-bearing equipment during lunge-stepping, but in their study, the subjects were active and only the muscle activation level was studied. Other aspects of biomechanics were not addressed, such as maximum peak angle and peak moment. Choosing the right type of load condition improves the exerciser’s results [19]. In addition, compared to the deep squat, the lunge squat requires more balance due to the movement of the center of gravity on the ground. The external force applied to the body also follows the changes in force, causing the mobilization of many muscles to maintain body stability [8]. Experienced fitness enthusiasts demonstrate greater muscular strength and better joint coordination, producing higher power and utilizing muscle force generation through joint coordination [25]. Whereas for fitness novices, muscle imbalance and poor body coordination, combined with a tendency to lean forward during the lunge, increases the shear force at the knee joint, increasing the risk of injury [26,27]. Fitness instructors should develop the most suitable lunge exercise program for fitness participants based on experience.

A large number of studies have focused on different exercise types, such as comparing biomechanical changes and muscle activation in the deep squat, single-leg squat, and lunge squat [4,5,8,10,18,24,28,29,30]. Some studies have focused on the effects of different postures, such as the degree of inclination of the dominant calf and lunge step length [22,24,31,32]. All of these studies used load weight fixing in these tests. In contrast, Bryan [9] and Nadzalan [7] did a longitudinal study using weights, comparing changes in the hip, knee, and ankle at different load intensities while focusing on young people. With the improvement of young people’s living standards and health awareness, an increasing number of males are concerned about their physical development [33] and they want to develop their muscles using scientific perspectives, relying not only on subjective experience in selecting exercise movements. However, novice athletes are inefficient and at risk of injury due to a lack of theoretical knowledge and irrational movement choices [34]. Therefore, the main purpose of this study was to investigate the kinematic and kinetic changes of the hip, knee, and ankle joints of male fitness beginners at 25% body weight (BW) and 50% BW with dumbbells, barbells, and weighted vests using OpenSim, and to provide appropriate guidance based on the results of the study. Therefore, three hypotheses are presented: (a) The three weight types have different degrees of influence on the kinematics and kinetics of the hip, knee, and ankle. (b) The influence of the weight types on the joint deepens as the intensity of the weight increases. (c) There are one or more appropriate weight types and intensity combinations due to the differences in the exercise effect of the different weight types.

## 2. Materials and Methods

### 2.1. Participants

Fourteen male fitness novices were recruited for this study (age: 22.7 ± 1.6 years, height: 1.72 ± 0.05 m, weight: 73.17 ± 0.02 kg, BMI: 24.89 ± 1.52 kg/m^2^, leg length: 0.86 ± 0.04 m, shoulder width: 0.41 ± 0.03 m). The dominant leg was identified by preference during kicking and the preferred limb was defined as the dominant limb. There were no lower limb diseases or injuries in the six months before testing. Participants did not eat for 2 h before the experiment and were prohibited from consuming any type of alcohol or caffeine for 24 h. They all wore the same style of shoes to avoid the effects of shoes. All participants understood the purpose and significance of the study, were informed of the testing procedures, and signed an informed consent form before experimental data collection. The Ethics Committee of Ningbo University Research Institute approved the experiment (No: RAGH202110283004.2).

### 2.2. Instruments

**Figure 1:**
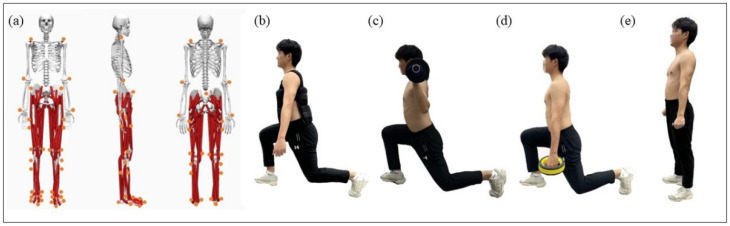
(**a**): Front, side, and back view of the subject’s reflective markers; (**b**): vest lunge pose; (**c**): barbell start pose; (**d**): dumbbell lunge pose; (**e**): lunge start pose.

An eight-camera Vicon motion capture system (Vicon Metrics Ltd., Oxford, UK) was used to capture motion trajectories. An embedded Kistler 3D force plate (Kistler, Switzerland) was used to record ground reaction forces simultaneously at 200 Hz and 1000 Hz. Before the experiment, subjects were asked to apply 41 reflective markers (diameter: 14 mm) to their bodies. The specific location points are outlined in Figure 1. The midpoint of the femur was defined as the central point of the greater trochanter and the epicondyle of the femur. The midpoint of the fibula was defined as the central point of the lateral condyle and the lateral ankle.

### 2.3. Procedures

Participants performed a 5 min warm-up on a stationary treadmill before performing a lunge squat. The leg length of each participant was measured using a metric ruler. Leg length was defined as the length from the anterior superior iliac spine to the medial tibial condyle. We defined stride length as 70% leg length and marked the corresponding stride length with tape at the start point and target stride point (on the force platform) as the lunge stride length for each participant.

Before the experiment began, participants underwent practice experiments for each weight type; subjects were instructed by related professional practitioners, and verbal instruction ensured familiarity with the movements without major problems. At the beginning of the experiment, the participants stood with their feet together at the starting point, and after hearing the command, they moved the dominant leg forward to the marked position. They then shifted their body weight in a downward direction and kept their trunk upright. In the end, the non-dominant leg moved forward and landed in a position parallel to the dominant leg. The body weight returned to the initial state and ended with the body upright and knees fully extended as the end of a full movement. We specified that a qualified movement required: (1) the torso to remain upright and perpendicular to horizontal, with the arms always on either side of the torso and without swaying; (2) the knee joint of the dominant leg to go no further forward than the toe and the lower leg perpendicular to the ground; (3) the knee joint of the non-dominant leg to be close to the ground but not touching the ground. When all three conditions were met, it was deemed a qualified action.

Participants performed a total of six sets of experiments, with each individual’s weight-bearing intensity and order of weight-bearing type determined by completely random selection. Six qualifying data were collected for each condition, from which three lunges were selected. After each set was completed, participants rested for 5 min to prevent fatigue. The lunge squat had six weighting situations with 25% and 50% BW of dumbbells, barbells, and weighted vests [9,24], as outlined in Figure 1. The barbell was kept in position behind the back and the dumbbells of the same weight were held by both the left and right upper limbs. Vest contact with the subject’s skin ensured that it was always fixed.

### 2.4. Data Collection and Processing

One lunge data period was defined as starting from the previous frame of the dominant leg contacting the force table to the end of the next stance position. The kinematic and kinetic data (including peak flexion angle (PFA), ROM, and peak moments, as well as center of pressure (COP)) of the hip, knee, and ankle joints in the sagittal planes were collected to analyze joint changes under different load conditions.

Each set of data for PFA, ROM, peak moment, and COP was normalized to 0–100%, whereas subjects’ body weight and weight after weight bearing were used to normalize joint moments. COP was normalized for leg length to eliminate the effect of height [35].

Marker trajectories and ground reaction forces were filtered by zero-latency fourth-order Butterworth low-pass filters at 12 Hz and 30 Hz, respectively. A threshold of 20 N was used, and ground reaction force data below 20 were rejected. The C3D file data were converted to formats recognized in OpenSim 4.3 (.mot and .trc) by Matlab R2018a (The MathWorks, MA, USA), and then imported into OpenSim for data processing [36]. The model employed in this experiment was adapted by Lu et al. [37] based on the open-source original model of the deep squat [38] to prevent muscles from crossing bones and causing higher joint mobility. The model was scaled using the subject’s marker point location and weight in a static calibration. The static weight of each marker was manually adjusted according to the root mean square (RMS) error value (less than 0.02) between the experimental and virtual markers in the model until it was adjusted to the appropriate position before applying the scaled model to the data calculation. The joint angles were calculated using the inverse kinematics (IK) calculation tool in OpenSim and the results were optimized using least squares to minimize the error between the experimental and virtual markers. The inverse dynamics (ID) algorithm was used to calculate the net moments of the hip, knee, and ankle joints. The ID tool performs inverse dynamic analysis by applying these data given the kinematics describing the movement of the model and perhaps a portion of the kinetics applied to the model. The ID tool solves the mathematical equations of force and acceleration of classical mechanics in an inverse dynamics sense to yield the net forces and torques at each joint which produce the movement [39].

### 2.5. Statistical Analysis

In this study, statistical parametric mapping was used to compare joint angles and moments, and one-dimensional statistical parametric mapping (SPM1D) was performed within the SPM1D package based on MATLAB. IBM SPSS Statistics 26 (IBM Corporation, Armonk, NY, USA) was used to analyze sagittal ROM, sagittal PFA, peak moments, and COP for different conditions in the *x*- and *y*-axes. Two-way repeated measures were used to analyze for significance. The significance level was set at *p* < 0.05.

## 3. Results

### 3.1. Hip, Knee, and Ankle Joint Angles

**Figure 2:**
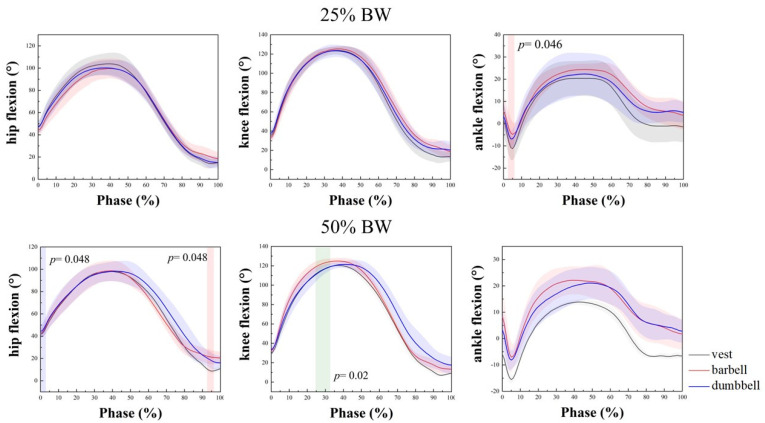
Joint angles and SPM1D results for hip, knee, and ankle at 25% BW and 50% BW. Red: indicates a significant difference between vest and barbell; green: indicates a significant difference between barbell and dumbbell. BW: body weight.

The kinematic joint results and SPM1D results for the hip, knee, and ankle joints at 25% BW and 50% BW are shown in Figure 2 and Table 1.

Two-way repeated measures analysis was performed to compare the effects on joint ROM and PFA at different load types and load intensities. The between-group (group) effects were 25% BW and 50% BW load intensity, and the within-group (type) effects were changes in joint angles due to vest, barbell, and dumbbell load types. There were no abnormal data as judged by box-line plots. The variance–covariance matrix of the dependent variable was equal for the interaction term group × type by Mauchly’s sphericity test (*p* > 0.05). For ROM and PFA in the hip joint, the group × type interaction was not statistically significant, and no significant differences were found between the main effects of group and type. ROM and PFA for vest, barbell, and dumbbell all had a decreasing trend at 50% BW compared to 25% BW. In Figure 2, in the results of SPM1D, a significant difference between the vest and barbell at 50% BW load intensity was observed in the rise and stand phase (*p* = 0.048). At ROM of the knee joint, the interaction of group × type was statistically significant, F (2, 16) = 4.744, *p* = 0.024. The separate effect of the group on the barbell was statistically significant (*p* = 0.038), and the barbell was significant at 25% BW and 50% BW (*p* = 0.038). At 50% BW, the barbell was significantly different from the dumbbell (*p =* 0.033), as shown in Table 1, and ROM increased with increasing intensity. In Table 1, PFA decreased with increasing intensity and use of the barbell decreased PFA the least, by 0.74°, whereas SPM1D results showed a significant difference between barbell and dumbbell during squatting at 50% BW deadlift strength (*p =* 0.02). Both barbell and dumbbell use increased ankle ROM, but the vest and the dumbbell increased ankle ROM more, by 1.44°. SPM1D found a significant difference between the vest and barbell at 25% BW deadlift strength when the foot touched the ground (*p =* 0.046). A significant difference in the main effect of weight types was found at PFA (*p =* 0.038). A significant difference was found between the vest and barbell (*p =* 0.022). In addition, 50% BW with the vest decreased by 3.23°, compared to 25% BW and 50% BW with the barbell which decreased by 2.01° and compared to 25% BW with dumbbells which increased by 0.08°. Overall, the barbell varied to a greater degree at the knee joint than the other two by intensity. Dumbbells varied less at the three joints than at the other two.

### 3.2. Hip, Knee, and Ankle Joint Moments

**Figure 3:**
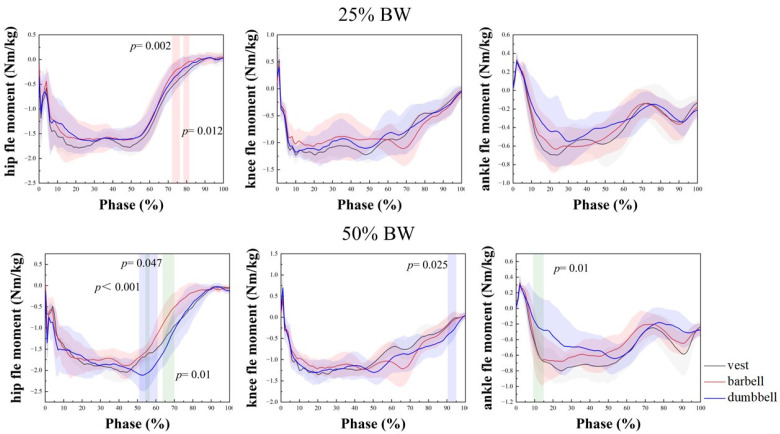
Joint moments and SPM1D results for hip, knee, and ankle at 25% BW and 50% BW. Red: indicates a significant difference between vest and barbell; green: indicates a significant difference between barbell and dumbbell; blue: indicates a significant difference between vest and dumbbell. BW: body weight.

The joint moments of the hip, knee, and ankle joints at 25% and 50% of body weight, PFA, and SPM1D results are shown in Figure 3 and Table 2.

As shown in Table 2, there was no significant difference in the hip group × type interaction, but there was a significant difference in the main effect of weight-bearing strength, F (1, 11) = 93.13, *p* < 0.001. Furthermore, 25% BW was 0.259 Nm/kg smaller than 50% BW at weight-bearing strength, a statistically significant difference (*p* < 0.001). At 25% BW, the peak barbell flexion moment was the smallest at 1.87 Nm/kg, followed by the dumbbell and the largest was the vest at 1.96 Nm/kg. Moreover, 50% BW increased the most over 25% BW with dumbbells at 0.4 Nm/kg and the least with the vest at 0.16 Nm/kg. In the SPM1D results in Figure 3, there was a significant difference between the vest and barbell rise and stand phase at 25% BW weight-bearing strength (*p =* 0.002, *p =* 0.012). At 50% BW weight-bearing strength, there was a significant difference between the barbell and dumbbell (*p =* 0.047, *p =* 0.01), and also between the vest and dumbbell (*p* < 0.001). On the knee joint, the main effect of weight-bearing strength was statistically significant, F (1, 11) = 9.058, *p =* 0.012. The largest increase at 50% BW over 25% BW was with the barbell, which increased by 0.13 Nm/kg from 1.46 to 1.59 Nm/kg, and the smallest was with the vest, which increased by 0.03 Nm/kg from 1.41 to 1.44 Nm/kg. In the results of SPM1D, there was a significant difference between the vest and the dumbbell in the standing phase at 50% BW (*p =* 0.025). At the ankle joint, the group × type interaction was significantly different for the ankle (*p =* 0.006), and analysis of the separate effects of group and type revealed a significant difference between the vest at 25% BW and 50% BW (*p =* 0.006). The vest increased most, from 0.79 to 0.98 Nm/kg, an increase of 0.19 Nm/kg; whereas the barbell and dumbbell both increased by 0.11 Nm/kg. At 50% BW, the vest was 0.98 Nm/kg, and the dumbbell was 0.81 Nm/kg; there was a significant difference between them (*p =* 0.023). In the SPM1D plot, it was shown that at 50% BW, there was a significant difference between the dumbbell and the barbell during the squat (*p =* 0.01). Overall, the barbell varied to a greater degree of variation in weight strength at the knee joint than the other two, and the dumbbell had relatively little overall variation at all three joints. The vest, on the other hand, showed the least variation in ROM at the hip joint.

### 3.3. The COP of the Lunge

**Figure 4:**
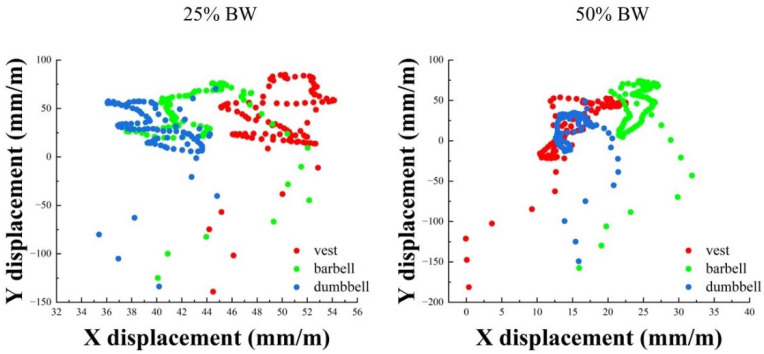
COP motion trajectory at 25% BW and 50% BW. BW: body weight.

The COP motion trajectory and *x*-axis offset range and *y*-axis offset at 25% BW and 50% BW are shown in Figure 4 and Table 3.

As shown in Figure 4, it can be seen that the overall offset range with the dumbbell was smaller at 25% BW and 50% BW, whereas the overall trajectory with the barbell was larger at 25% BW. As shown in Table 3, in the offset range of the COP x-axis, there was no significant difference in the group × type interaction, but there was a significant difference in the main effect of weight types, F (2, 16) = 3.689, *p =* 0.048, and there was a statistically significant difference between barbell and dumbbell (*p =* 0.027). The offset range of both vest and dumbbell increased and the vest increased the most with 4.78 mm/m. In the *y*-axis offset, there was no significant difference in the group × type interaction. There was a significant difference in the main effect of weight strength, F (1, 9) = 25.077, *p =* 0.001. Additionally, 25% BW had a statistically significant difference of 2.453 mm.s/m smaller offset than 50% BW at weight strength (*p =* 0.001). The *y*-axis was the same as the x-axis, and both had the largest increase in vest offset of 3.08 mm.s/m.

## 4. Discussion

The purpose of this study was to investigate changes in lower limb kinematics and kinetics of male fitness novices using dumbbells, barbells, and weighted vests at 25% BW and 50% BW weights and to provide appropriate exercise choices and fitness instructions accordingly. This study found that, as strength increased, the kinematics of the barbell at the knee joint was more influential than the other two by studying different weight types and intensities, whereas the SPM1D was also used to analyze the process of joint angles and moments in this study compared to previous studies. All three weight types increased the joint moment with the increase in weight strength, and the increase in all three types was significant at the hip and knee joints, with the dumbbell increasing the most at the hip, the barbell increasing the most at the knee, and only the vest increasing significantly at the ankle joint. For COP, dumbbells showed the least variation whereas barbells decreased the COP. Weight type and intensity had a greater effect on kinetics than on kinematics, and SPM1D showed more significant results at 50% BW, which confirms our first and second hypotheses.

During the lunge phase, our study found that the knee joint had the greatest ROM and the most PFA. The ROM of the knee increased with increasing load intensity, with an average increase of 5.06° for all three load types, which is consistent with the findings of Bryan et al. [9]. The largest increase in ROM of the knee was 11.15° during barbell load conditions. In addition, a study by Danielle et al. [40] also showed an effect between greater knee flexion angle and knee joint stress during the anterior lunge. According to Zellmer et al. [41], it was reported that greater knee flexion could also lead to a tendency for the knee to translate forward. Peak kneel joint stress, quadriceps muscle strength, knee moment, knee flexion, and ankle dorsiflexion were greater when the knee was panned in front of the toes during the lunge. Keeping the knee behind the toes minimizes the stress on the patellar tendon. To reduce patellar forces, Escamilla et al. [27] suggested to be cautious about performing a long-step lunge squat. In addition to this, the anterior lunge has been shown to be part of an effective exercise program for the treatment of knee joint pain [42]. Based on the data, it is easy to see that dumbbells have a small and variable effect on the ROM of the knee relative to vests and barbells.

During the squat, we also observed a difference in joint ROM between the load cases, with SPM1D results indicating a significant difference between barbell and dumbbell at 50% BW for the knee joint. This difference may be related to the height of the entire center of gravity position. Compared to the dumbbell, the barbell’s shoulder-centered high center of gravity can be difficult for novices with less physical stability, and the higher the center of gravity, the more unstable it is. Several studies have demonstrated that load weight has little effect on joint mobility [14,24]. This study found some variation at the knee joint, and this finding of the load type effect on joint ROM was through a mixture of SPM1D and load type and intensity. Changes in the process have been reported infrequently in prior studies employing this statistical methodology. At the ankle joint, we found a significant change between the vest and the barbell at 25% BW, as seen in contact with the ground and progression from plantarflexion to dorsiflexion. There was also a significant difference between the vest and barbell on the ankle PFA. According to Guilherme et al. [43], in their study, the dynamic ankle dorsiflexion range may be changed by neuromuscular strategies induced by factors outside of the passive ankle dorsiflexion range. For example, factors of hip and knee muscle weakness may be associated with an increased demand for plantar flexors. When the hip and knee muscles are weak, the lower leg and foot muscles compensate by carrying the additional load to be able to stand. Likewise, if the knee flexion is small, this indicates the presence of other underlying factors such as quadriceps weakness and limited ankle dorsiflexion range [44]. In general, the ROM with dumbbells is smaller and less variable than the other two load types, whereas overall stability is better for beginners. In contrast, those who already have the relevant basic accumulation of exercise and want to make the exercise more effective can choose the barbell method of weight bearing because the ROM and PFA at the knee joint are large and may be more sensitive to the weight.

In terms of moments, we observed that the hip joint moment was the greatest, followed by the knee joint and, finally, the ankle joint. The results of SPM1D showed that there was a significant difference between the weight types at the hip joint after reaching the lowest point and then rising at both weights. We think that this is related to the position of the center of gravity. At high load intensities, there was a significant difference between the dumbbell and the other two load types because the dumbbell load was lower than the hip position at the sides of the body. The lower the body’s center of gravity, the greater the joint moment, as confirmed by the data at 50% BW. The average increase in the joint moment at 50% BW over 25% BW was 0.259 Nm/kg at the hip, 0.087 Nm/kg at the knee, and 0.139 Nm/kg at the ankle. The hip showed the greatest rise, followed by the ankle and, finally, the knee. These results confirm that the forward lunge is a hip-dominant movement and that increasing external loading has the greatest effect on the hip moment and the least on the knee joint. This varying degree of increase is consistent with the findings of Bryan [9], who argued that the knee joint has a greater range of motion than both the hip and the knee, and the examination of linear and quadratic trends in the net joint moment of the ankle suggests that the ankle may have reached the point of the maximum contribution. However, he also indicated that stronger external loading would be required to substantiate the findings. Farrokhi et al. [22] did not statistically compare hip, ankle, and knee joint dynamics. Their descriptive statistics and graphical data also showed that the forward lunge is a hip extensor-dominated movement. All of these studies indicate that the hip contributes more to the kinematics of the anterior lunge than the knee and ankle.

Bryan explained that the hip, knee, and ankle joints contribute to changes in the lunge accordingly. As the intensity of the load increases, little changes in the upper extremities or trunk, such as subjects changing the way they hold the dumbbell and moving it to a more forward position, may occur during the experiment. This phenomenon produces an effect comparable to the trunk forward lunge. The trunk forward lunge shifts the total body weight forward, and the corresponding forward movement of the barbell, dumbbell, and vest causes more mass to move forward, which may increase the contribution of the hips and ankles. Other studies have similarly pointed out that [31] as the anterior limb tibia-ground angle decreases, the ROM of the anterior knee becomes larger, and the flexion moment at the hip increases. Paul [23] suggested that due to the greater hip joint moment in the front lunge, it is more effective to choose the forward lunge over the single-leg deep squat and the back lunge if one wants to exercise the hip extensors. However, the forward shift of body weight may not be enough to ensure a meaningful increase in the hip extensors, allowing more muscle recruitment to occur in the other muscles, as mentioned by Farrokhi [22]. As seen by our data, at the hip joint, the joint moment when using the barbell at 25% BW was the smallest, followed by the dumbbell. However, the dumbbell varied greatly with increasing weight. At the knee joint, the vest produced the smallest joint moment, and at the ankle joint the dumbbell had the smallest. If we choose to exercise the hip and knee muscles more, we can choose large-weight dumbbells. If we want less load on the knee joint, we can choose a small-weight vest as the external load carrier.

For the COP displacements, it is difficult to obtain a specific understanding of the data from the point plots. The overall movement of the dumbbells was hard to distinguish, therefore we quantified the *x*-axis and *y*-axis data. The *x*-axis represents the displacement of the lateral COP, and the *y*-axis represents the displacement of the anterior and posterior aspects. We processed the *y*-axis data to obtain the overall offset of the *y*-axis. In the *x*-axis direction, both the vest and dumbbell increased in range with increasing weight, and there was a significant difference between the barbell and dumbbell, with an average increase of 4.78 mm/m with the vest and 3.49 mm/m with the dumbbell, whereas the *y*-axis offset was affected by the weight strength, with a larger offset of 2.453 mm.s/m for 50% BW than for 25% BW weight strength. The vest increased by 3.08 mm.s/m, and the dumbbell increased by 1.65 mm.s/m. This result was expected because the overall muscle strength was weak under heavy weights, and the body could not be easily controlled to complete the prescribed movements. The barbell, however, showed a decrease, as did the *y*-axis. We cannot explain this unexpected result specifically, probably because the upper limbs share some of the weight. However, for a wide range of displacements, Hsue et al. [35] suggested that excessive inclination leads to excessive COM and COP separation, requiring greater active postural control and more energy to counteract the increased joint moment, which would lead to loss of balance and thus accidents. Combining the above findings, it is easy to corroborate the third hypothesis that each of the three weight types has its benefits, thus selecting the correct type of weight according to the specific physical qualities of the exerciser is important.

Our study has several limitations. First, we only studied male populations; based on the relatively weak strength of female novices, we did not examine the performance of female populations in the lunge. We did not reflect the gender difference. Secondly, we only selected 25% BW and 50% BW and did not explore the results of adding weights in between or exploring the results of weights above 50% BW. Future studies should focus more on the diversity of the population, variation of the backward lunge under different weight conditions, and the effect of learning effects on results when performing different weight orders. Future studies will be more careful.

## 5. Conclusions

We examined different types of weight-bearing exercises. The strength of the load caused the kinematics and kinetics of the barbell to vary more at the knee joint. The kinematics of the dumbbell were less variable at all three joints, whereas the kinetics varied the most at the hip joint. The vest had the greatest effect on the joint moment at the ankle joint, whereas the joint moment changed the least at the hip and knee joints and the kinematics changed the most. All three weight-bearing types had larger joint moments with increasing intensity. These results are consistent with our expected hypothesis. One of the important findings was that all three types of weights have their own advantages for the fitness enthusiast, and training specificity and desired outcomes are important in the selection of weight-bearing exercise. Dumbbells seem to be more suitable for male beginners using the lunge exercise because of the low impact on the knee joint and high stability. For those who want to strengthen the hip and knee muscles, they can choose dumbbells with a high weight. For individuals that have accumulated experience, they can choose barbells as the main lunge exercise equipment. For people suffering from corresponding knee and knee joint pain, a small-weighted vest lunge exercise may be the best choice for rehabilitation and injury avoidance.

## Figures and Tables

**Table 1:** Sagittal ROM and PFA of the hip, knee, and ankle at 25% BW and 50% BW.

		25% BW (Mean ± SD)		50% BW (Mean ± SD)	
		ROM	PFA	ROM	PFA
vest (°)	hip	90.04 ± 8.26	101.92 ± 9.79	88.38 ± 2.95	95.97 ± 2.57
	knee	108.98 ± 7.33	124.33 ± 3.93	110.52 ± 8.24	122.37 ± 2.37
	ankle	34.13 ± 5.66	23.51 ± 8.73 ^d^	33.73 ± 5.02	20.28 ± 7.01 ^d^
barbell (°)	hip	83.86 ± 12.26	101.62 ± 9.04	81.23 ± 12.68	100.23 ± 9.48
	knee	103.58 ± 6.19 ^b^	125.81 ± 2.63	114.73 ± 4.21 ^ab^	125.07 ± 2.80
	ankle	32.72 ± 2.98	25.50 ± 3.51 ^d^	32.80 ± 4.79	23.49 ± 6.75 ^d^
dumbbell (°)	hip	86.35 ± 5.47	100.23 ± 6.69	84.26 ± 10.00	99.70 ± 9.43
	knee	105.42 ± 3.56	123.31 ± 6.96	107.90 ± 3.32 ^a^	122.00 ± 4.65
	ankle	29.96 ± 7.12	21.84 ± 10.44	31.40 ± 5.78	21.92 ± 7.15

Note: ^a^ indicates significance *p* < 0.05 for the same weight; ^b^ indicates significance *p* < 0.05 for different weights for the same loading type; ^d^ indicates the significance of the main effect of type *p* < 0.05.

**Table 2:** Peak joint moments of hip, knee, and ankle at 25% BW and 50% BW.

		25% BW (Mean ± SD)	50% BW (Mean ± SD)
vest (Nm/kg)	hip	−1.96 ± 0.14 ^c^	−2.12 ± 0.24 ^c^
	knee	−1.41 ± 0.11 ^c^	−1.44 ± 0.17 ^c^
	ankle	−0.79 ± 0.15 ^b^	−0.98 ± 0.19 ^ab^
barbell (Nm/kg)	hip	−1.87 ± 0.32 ^c^	−2.09 ± 0.32 ^c^
	knee	−1.46 ± 0.13 ^c^	−1.59 ± 0.17 ^c^
	ankle	−0.78 ± 0.18	−0.89 ± 0.22
dumbbell (Nm/kg)	hip	−1.90 ± 0.29 ^c^	−2.30 ± 0.30 ^c^
	knee	−1.47 ± 0.20 ^c^	−1.58 ± 0.12 ^c^
	ankle	−0.70 ± 0.21	−0.81 ± 0.20 ^a^

Note: ^a^ indicates significance *p* < 0.05 for the same weight; ^b^ indicates significance *p* < 0.05 for different weights for the same load type; ^c^ indicates significance *p* < 0.05 for the main effect of the group.

**Table 3:** COP *x*-axis offset range and *y*-axis offset at 25% BW and 50% BW.

	25% BW (Mean ± SD)		50% BW (Mean ± SD)	
	*x*-axis (mm/m)	*y*-axis (mm.s/m)	*x*-axis (mm/m)	*y*-axis (mm.s/m)
vest	31.07 ± 9.00	5.35 ± 1.76 ^c^	35.85 ± 9.13	8.43 ± 1.94 ^c^
barbell	38.08 ± 10.43 ^d^	6.99 ± 3.37 ^c^	35.37 ± 5.47 ^d^	4.84 ± 1.68 ^c^
dumbbell	28.26 ± 4.59 ^d^	4.88 ± 2.54 ^c^	31.75 ± 4.82 ^d^	6.53 ± 2.81 ^c^

Note: ^c^ indicates significance *p* < 0.05 for the main effect of the group. ^d^ indicates the significance of the main effect of type *p* < 0.05.

## Data Availability

The data that support the findings of this study are available upon reasonable request from the corresponding author. The data were not publicly available because of privacy or ethical restrictions.
